# In-vivo glenohumeral translation and ligament elongation during abduction and abduction with internal and external rotation

**DOI:** 10.1186/1749-799X-7-29

**Published:** 2012-06-28

**Authors:** Daniel F Massimini, Patrick J Boyer, Ramprasad Papannagari, Thomas J Gill, Jon P Warner, Guoan Li

**Affiliations:** 1 Massachusetts General Hospital, Harvard Medical School, Bioengineering Laboratory, GRJ-1215, 55 Fruit Street, Boston, MA, 02114, USA; 2 Massachusetts Institute of Technology, Department of Mechanical Engineering, 77 Mass Ave, Cambridge, MA, 02139, USA

## Abstract

**Study Design:**

Basic Science. To investigate humeral head translations and glenohumeral ligament elongation with a dual fluoroscopic imaging system.

**Background:**

The glenohumeral ligaments are partially responsible for restraining the humeral head during the extremes of shoulder motion. However, in-vivo glenohumeral ligaments elongation patterns have yet to be determined. Therefore, the objectives of this study were to 1) quantify the in-vivo humeral head translations and glenohumeral ligament elongations during functional shoulder positions, 2) compare the inferred glenohumeral ligament functions with previous literature and 3) create a baseline data of healthy adult shoulder glenohumeral ligament lengths as controls for future studies.

**Methods:**

Five healthy adult shoulders were studied with a validated dual fluoroscopic imaging system (DFIS) and MR imaging technique. Humeral head translations and the superior, middle and inferior glenohumeral ligaments (SGHL, MGHL, IGHL) elongations were determined.

**Results:**

The humeral head center on average translated in a range of 6.0mm in the anterior-posterior direction and 2.5mm in the superior-inferior direction. The MGHL showed greater elongation over a broader range of shoulder motion than the SGHL. The anterior-band (AB)-IGHL showed maximum elongation at 90° abduction with maximum external rotation. The posterior-band (PB)-IGHL showed maximum elongation at 90° abduction with maximum internal rotation.

**Discussion:**

The results demonstrated that the humeral head translated statistically more in the anterior-posterior direction than the superior-inferior direction (*p* = 0.01), which supports the concept that glenohumeral kinematics are not ball-in-socket mechanics. The AB-IGHL elongation pattern makes it an important static structure to restrain anterior subluxation of the humeral head during the externally rotated cocking phase of throwing motion. These data suggest that in healthy adult shoulders the ligamentous structures of the glenohumeral joint are not fully elongated in many shoulder positions, but function as restraints at the extremes of glenohumeral motion. Clinically, these results may be helpful in restoring ligament anatomy during the treatment of anterior instability of the shoulder.

## Background

The shoulder (glenohumeral) joint has the widest range of motion of all major joints, due to its limited articular constraint and lax capsuloligamentous structures. Its stability is afforded through the combined effect of articular geometry, capsuloligamentous restraint and dynamic compression through the rotator cuff. The capsuloligamentous structures have defined thickenings called ligamentous bands [1-3] at defined regions of the joint capsule. Prior in-vitro ligament cutting studies [3,4] have given evidence to the likely function of these ligament bands. Some in-vitro investigations have analyzed the role of the capsuloligamentous structures with simulated muscle actions [5-7] and structural/failure analysis [8-10] have been performed to provide insight into the stabilizing role of the joint capsule. Additionally, several cadaveric studies have reported on humeral head translations [5,11,12] during simulated shoulder motions.

While these in-vitro investigations have provided data on the biomechanical response of the shoulder under externally applied loads, the in-vivo ligament kinematics under physiological loading conditions have remained unclear. Most attempts to gain insight into the in-vivo function of the capsuloligamentous structures have used radiographic analysis of kinematics including conventional radiography [13,14], computed tomography (CT) [15,16] and magnetic resonance imaging (MRI) [17-19]. Few studies have attempted to investigate the anterior capsuloligamentous structures elongation pattern using radiopaque markers [4], and electromagnetic tracking [20]. However, the in-vivo glenohumeral ligamentous bands elongation patterns during functional shoulder motion have not been reported.

Therefore, the objectives of this study were to 1) quantify the in-vivo humeral head translations and glenohumeral ligament elongations during functional shoulder positions, 2) compare the inferred glenohumeral ligament functions with previous literature and 3) create a baseline data of healthy adult shoulder glenohumeral ligament lengths as controls for future studies. Humeral head translations and glenohumeral ligament lengths were determined with a combined dual fluoroscopic imaging system (DFIS) and MR imaging technique. DFIS is a validated non-invasive three dimensional musculoskeletal modeling technique that combines pairs of fluoroscopic images with bone models segmented from high resolution MRI [21-23].

## Methods

This study and the use of human subjects were approved by our institution’s IRB, and informed consent was obtained from all subjects before participating. Each subject underwent a clinical shoulder exam, including range of motion, subjective rotator cuff strength and test of apprehension for instability. As determined by a fellowship trained shoulder orthopaedic surgeon, all subjects had a clinically ‘normal’ uninjured / healthy result for all tests. Each subject self reported themselves as healthy and without pain or history of trauma to their shoulders. In total, five healthy adult male shoulders (2 left and 3 right) were studied. The average age of the subjects was 26 ± 4 years of age and the choice of left or right shoulder was randomly made by the subjects themselves.

**Figure 1:**
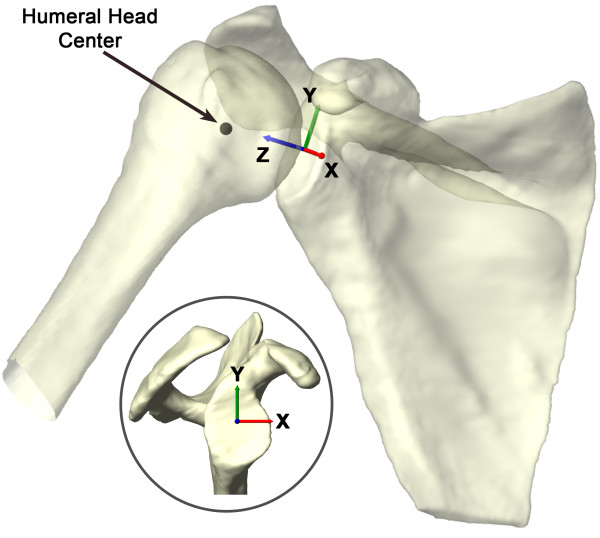
**“Shoulder joint surface model”** A typical shoulder joint model constructed from a patient specific MRI shown with the glenoid coordinate system definition used in this study.

**Figure 2:**
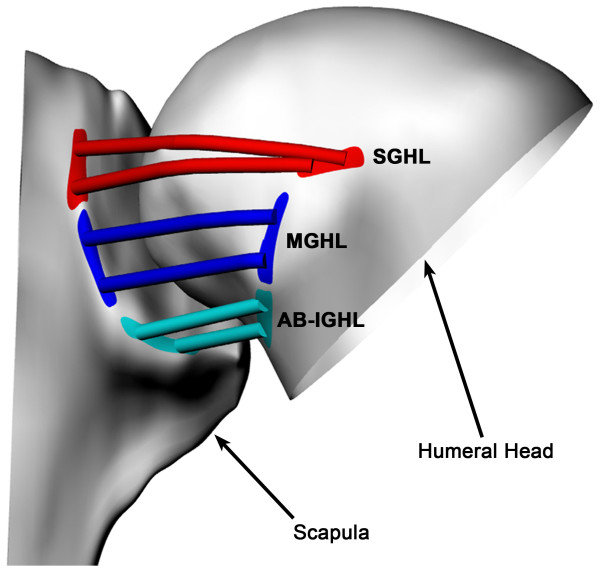
**“Glenohumeral ligaments on shoulder model”** A typical shoulder joint with the superior, middle and anterior-band of the inferior glenohumeral ligament (SGHL, MGHL and AB-IGHL) insertion areas shown. The posterior-band of the inferior glenohumeral ligament (PB-IGHL) is not shown for clarity.

Each shoulder was scanned with a 1.5 Tesla magnet (GE, Milwaukee, WI) using a Fast Image Employing Steady-state Acquisition (FIESTA) sequence. The MR scan created a cubic viewing volume of approximately 16 cm per side. Parallel sagittal plane images of the shoulder at 1.0mm intervals were acquired with a resolution of 512 by 512 pixels. The bony contours of the humeral head and scapula were manually outlined within 3D modeling software (Rhinoceros®, Robert McNeel & Associates, Seattle, WA). These outlines were then used to create 3D surface models of the shoulder joint (Figure 1). Based on prior anatomical work [1,2,7] the origin and insertion of the ligamentous components of the glenohumeral joint capsule were assigned based on each subject specific MRI. The insertion areas of the superior, middle and inferior glenohumeral ligaments (SGHL, MGHL and IGHL) were defined on the bony models (Figure 2) by a fellowship trained shoulder orthopaedic surgeon. The IGHL was divided into the anterior band (AB-IGHL) and posterior band (PB-IGHL).

**Figure 3:**
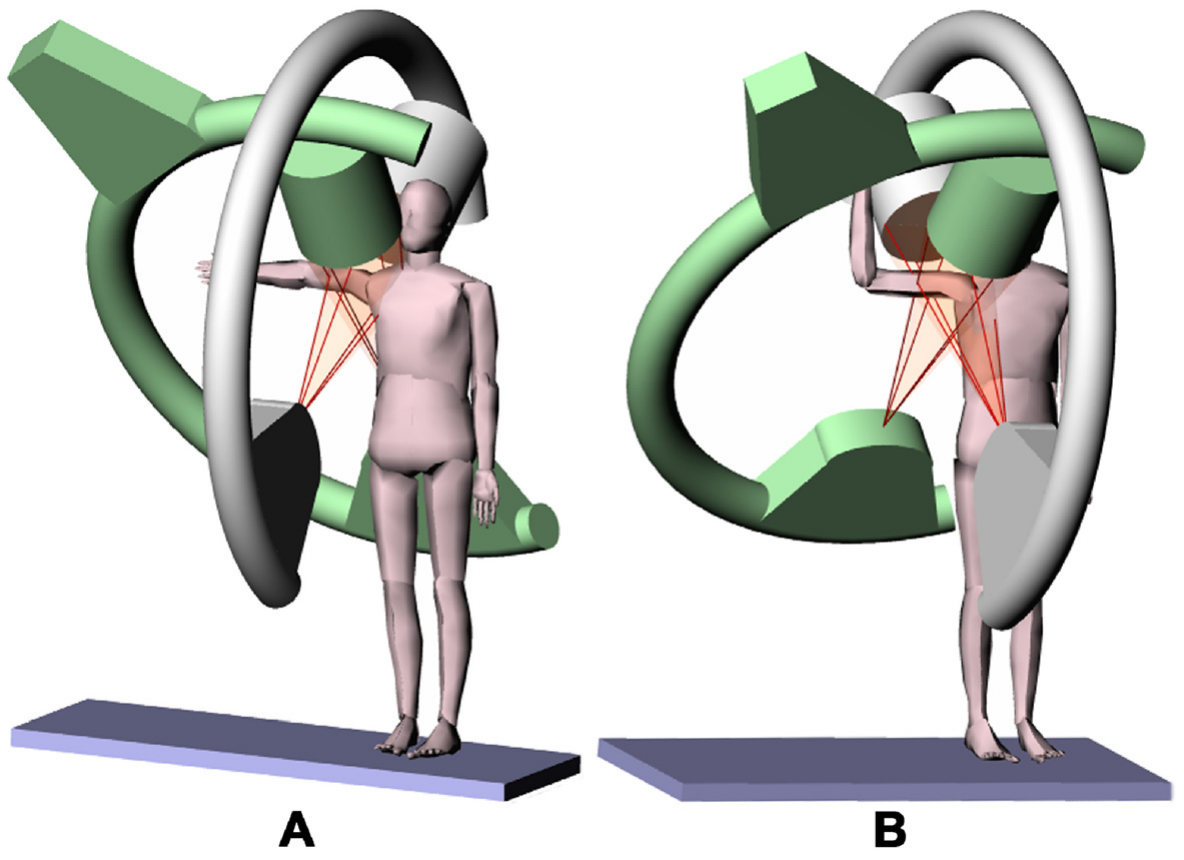
**“Testing environment of DFIS”** Dual fluoroscopic imaging system (DFIS) showed with (A) a subject in 90° abduction (B) a subject in 90° abduction with 90° external rotation.

After MR scanning, each subject was positioned inside a DFIS to capture in-vivo quasi-static shoulder motion. Two fluoroscopes (OEC 9800, GE, Milwaukee, WI) were positioned so that the subject could stand upright and the shoulder be simultaneously positioned in the imaging zones of both fluoroscopes (Figure 3).

**Figure 4:**
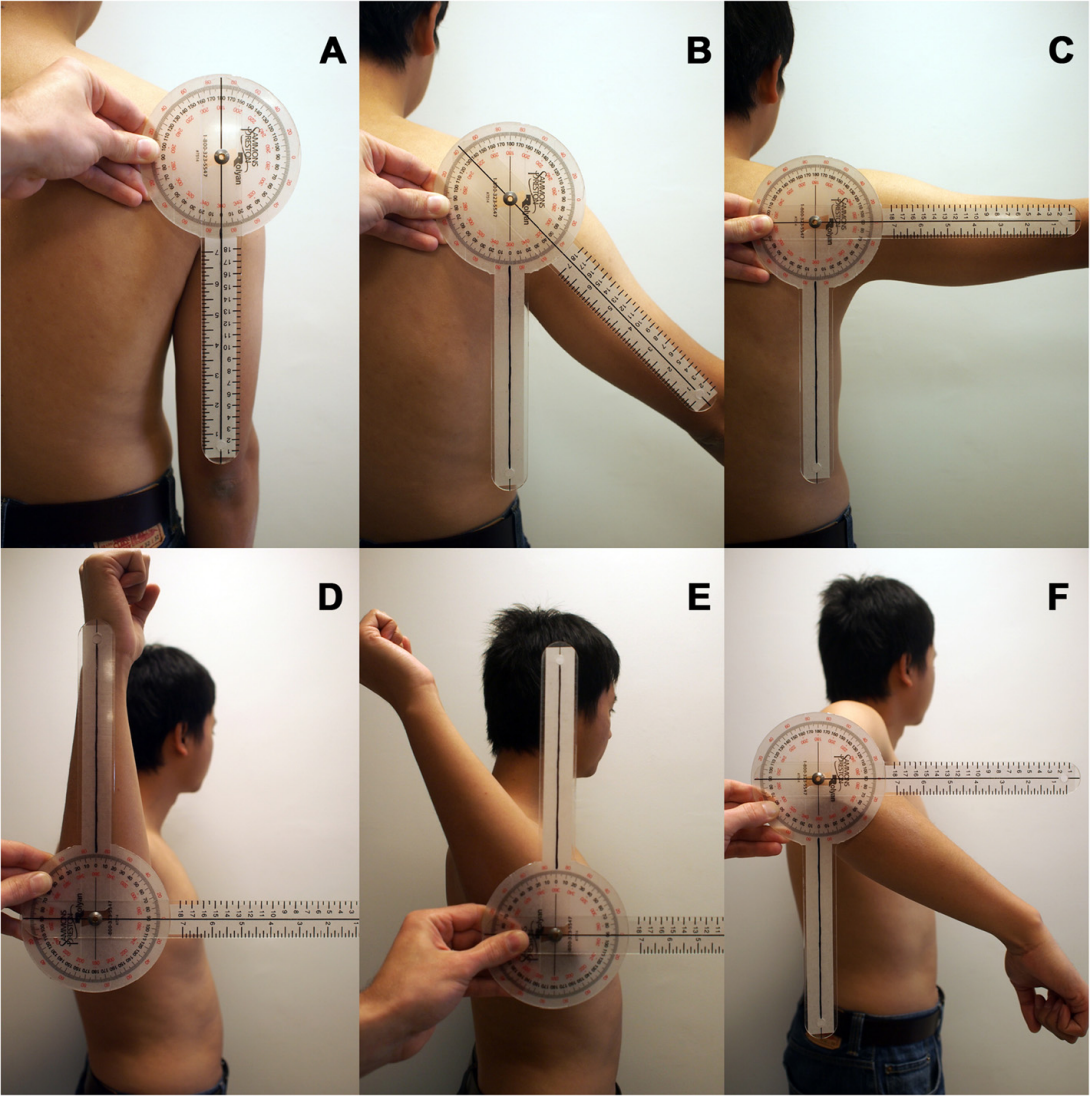
**“Shoulder angles in the coronal plane measured with a gonimeter”** (A) 0° abduction neutral rotation (B) 45° abduction neutral rotation (C) 90° abduction neutral rotation (D) 90° abduction with 90° external rotation (E) 90° abduction with maximum active external rotation (F) 90° abduction with maximum active internal rotation.

The shoulder was imaged in neutral rotation sequentially at 0°, 45° and 90° abduction (Figure 3A) while the subject stood in a relaxed position. During these positions, the elbow angle was maintained at 0°. In this study, the abduction angle was measured in the coronal plane and with respect to the vertical. A goniometer was used to determine the shoulder abduction and rotation angles (Figure 4). With the shoulder being maintained at 90° abduction the elbow was flexed to 90° and the arm externally rotated 90° around the longitudinal axis of the humerus and imaged by both fluoroscopes (Figure 3B). The subject was then asked to maximally externally rotate their shoulder similar to the starting position of throwing a baseball. Lastly, the shoulder was actively rotated to maximum internal rotation, while the arm was maintained at 90° abduction with an elbow angle of 90°. In total, six shoulder positions were tested (Figure 4).

**Figure 5:**
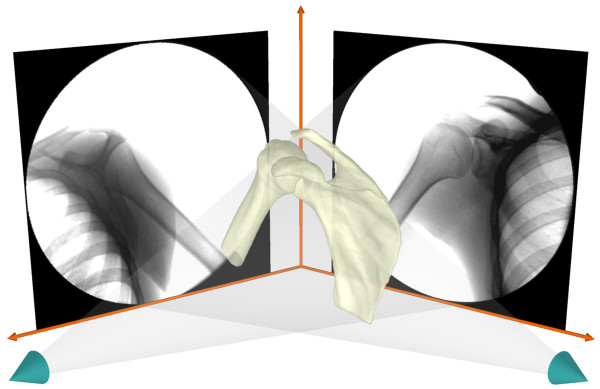
**“Computer environment of DFIS”** A virtual computer generated dual fluoroscopic imaging system (DFIS) in Rhinoceros 3D modeling software with a shoulder joint model positioned to reproduce the kinematics on the fluoroscopic images.

The fluoroscopic images and bones models were imported into a computer environment (Rhinoceros®, Robert McNeel & Associates, Seattle, WA) and each in-vivo shoulder pose manually reconstructed [21-23] (Figure 5). Each subject’s kinematic bone pose series was used to determine the motion of the humeral head center and ligament bands elongations. To quantify the motion of the humeral head, a sphere was fit to the articular geometry and the center taken as the center of the humeral head. The glenoid coordinate system was defined by a superior-inferior Y-axis between the most superior and inferior aspects of the glenoid rim. The coordinate system origin was placed at the midpoint of this axis on the glenoid surface. The X-axis was defined perpendicular to the Y-axis in the direction of the most anterior and posterior aspects of the glenoid rim (Figure 1). The Z-axis was normal to the X-Y plane, pointing towards the humeral head center. Humeral head translations were defined as the location of the humeral head center relative to the glenoid coordinate system. The humeral head center at 0° abduction was used as the reference position. The translations of the humeral head center along the anterior-posterior, superior-inferior, and medial-lateral directions are reported.

At each shoulder position tested, the area centroids of insertions of each glenohumeral ligament were connected using a wrapping curve around the humeral head surface (Figure 2). The length of the curve was measured to represent the ligament length at that in-vivo shoulder position. In this way, the SGHL, MGHL, AB-IGHL and PB-IGHL lengths were determined for each in-vivo shoulder pose. The ligament length at 0° abduction was used as the reference to calculate the glenohumeral ligament elongation for each ligamentous band. The glenohumeral ligaments functions were inferred from shoulder positions that exhibited the greatest relative ligament elongations based on a quote by Werner et al. that “the glenohumeral joint capsule provides passive stability at the extremes of glenohumeral motion [24].”

A repeated-measures analysis of variance (ANOVA) followed by a post-hoc Newman-Kuels test was used to detect which shoulder position (independent variable) had a significant effect on the humeral head translation (dependent variable). The X, Y, and Z translations were investigated independent of each other. To determine if the humeral head translated more in the anterior-posterior than superior-inferior direction, a Brown and Forsythe’s test on the variance of the motion relative to the 0° abduction position was performed. Ligament band lengths (dependent variable) as a function of shoulder position (independent variable) were determined statistically different with an ANOVA followed by a post-hoc Newman-Kuels test. The statistically significant difference was set at *p* < 0.05.

## Results

### Humeral head center translation

**Figure 6:**
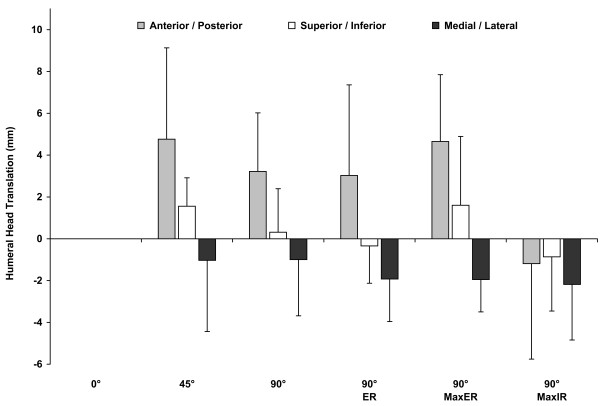
**“Humeral head translations”** Translations of the humeral head center relative to the glenoid at the shoulder positions tested. The humeral head center location at 0° abduction was used as the reference position. The translations of the humeral head center along the anterior-posterior, superior-inferior, and medial-lateral directions are reported.

As the abduction angle increased from 0° to 45°, the humeral head translated anteriorly on the glenoid to 4.8 ± 4.4mm (Figure 6). At 90° of abduction, the anteriorly translated humeral head was slightly reduced to 3.2 ± 2.8mm. Adding 90° of external rotation did not show noticeable change in the anterior position of the humeral head. At maximum external rotation, the humeral head translated anteriorly to 4.7 ± 3.2mm. Then at 90° abduction with maximum internal rotation, a posterior translation to −1.2 ± 4.6mm of the humeral head was observed. The humeral head center in the anterior-posterior direction was in a statistically different location relative to the glenoid between the shoulder positions of 45° abduction and 90° abduction with maximum internal rotation (p = 0.025) and between 90° abduction with maximum external rotation and 90° abduction with maximum internal rotation (p = 0.021).

As the abduction angle increased from 0° to 45°, the humeral head translated superiorly to 1.6 ± 1.4mm. At 90° of abduction, the humeral head translated inferiorly to 0.3 ± 2.1mm, and then with 90° of external rotation a further inferiorlization of the humeral head to −0.3 ± 1.8mm was observed. At maximum external rotation, the humeral head translated superiorly to 1.6 ± 3.3mm. Lastly, at 90° abduction with maximum internal rotation, the humeral head translated inferiorly to −0.9 ± 2.6mm. No statistical significance was detected in superior-inferior humeral head positioning among all tested shoulder poses.

At all positions of the shoulder that were tested, the humeral head center translated in an average range of −2.2mm with an average compression towards the glenoid surface by about −1.6mm compared to that at 0° abduction (Figure 6). At 45° of abduction, the humeral head center compressed toward the glenoid to −1.0 ± 3.4mm and at 90° abduction with 90° external rotation, the humeral head translated towards the glenoid surface to −1.9 ± 2.0mm. No statistical significance was detected in compression / distension of the humeral head center relative to the glenoid among all tested shoulder positions.

In all tested shoulder poses, for all translations of the humeral head center relative to the 0° abduction neutral rotation position of the shoulder, the average anterior-posterior translation range was 6.0mm with a variance of ±4.20mm. The average superior-inferior translation range was 2.5mm with a variance of the ±2.34mm. A Brown and Forsythe’s test of variance indicated that the amount of translation in the anterior-posterior (±4.20mm) direction was statistically different from the amount of translation in the superior-inferior (±2.34mm) direction (p = 0.01).

### Glenohumeral Ligament Lengths

**Figure 7:**
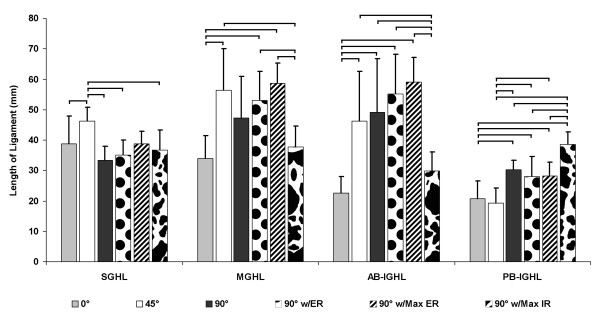
**“Glenohumeral ligament measured lengths”** The average measured lengths of the superior, middle, anterior-band and posterior-band of the inferior glenohumeral ligaments (SGHL, MGHL, AB-IGHL and PB-IGHL). Significant differences between ligaments length as a function of shoulder position have been denoted with a horizontal bracket.

**Figure 8:**
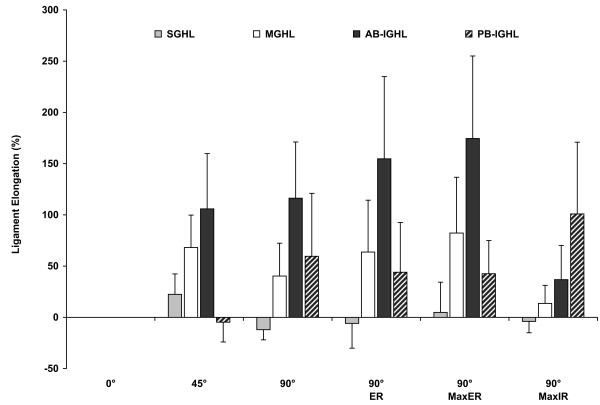
**“Glenohumeral ligament elongations”** Ligament elongations of the SGHL, MGHL, AB-IGHL and PB-IGHL are reported for the shoulder positions tested. The ligament length at 0° abduction was used as a reference to calculate ligament elongation.

The average ligamentous bands length of the SGHL, MGHL, AB-IGHL and PB-IGHL as a function of shoulder position are shown in Figure 7. The measured ligament lengths were used to calculate the relative elongation of each ligament band relative to its length at 0° abduction (Figure 8). Statistically significant differences between ligaments lengths as a function of shoulder position are denoted in the figure (Figure 7).

### Glenohumeral Ligament Elongations

The glenohumeral ligaments demonstrated different elongation patterns with each tested shoulder position (Figure 8). The SGHL length increased 22.5 ± 19.9% from 0° to 45° abduction. As the abduction angle increased to 90°, the SGHL elongation reduced to −12.1 ± 9.9% compared to that at 0° abduction. External rotation of the shoulder increased the SGHL length. At 90° abduction with maximum external rotation, the SGHL was elongated to 4.9 ± 29.5%, whereas, maximum internal rotation reduced the SGHL length to −3.9 ± 11.1%.

The MGHL length increased with abduction and with external rotation of the shoulder. At 45° abduction, the MGHL increased its length 68.1 ± 31.7%. The MGHL length was slightly reduced to 40.3 ± 32.0% at 90° of abduction. With 90° of external rotation, the MGHL length was increased to 63.7 ± 50.6%. At maximum external rotation, the MGHL elongation reached 82.2 ± 54.4%. However, with maximum internal rotation, the MGHL length was reduced to 13.6 ± 17.7% compared to that at 0° abduction.

The AB-IGHL length sharply increased to 105.9 ± 53.9% with abduction to 45° compared to that at 0° abduction. Thereafter, the AB-IGHL continued its length elongation with abduction and with external rotation. At 90° abduction with maximum external rotation, the AB-IGHL length increased to 174.6 ± 80.5%. However, with maximum internal rotation at 90° abduction, the AB-IGHL length was only increased to 36.8 ± 33.4%.

The PB-IGHL length was not significantly changed at 45° abduction compared to 0° (Figure 8). However, at 90° abduction, the elongation of the PB-IGHL increased to 59.6 ± 61.5%. Its elongation was slightly reduced with external rotation and was maintained at nearly 43% in both 90° external rotation and maximum external rotation. With maximum internal rotation of the shoulder, the PB-IGHL elongated to 100.9 ± 70.0% compared to that at 0° abduction.

## Discussion

Knowledge of shoulder biomechanics, such as glenohumeral kinematics, rotator cuff and ligament functions, are instrumental for successful surgical treatment of shoulder pathology. Currently, in-vitro cadaveric experiments have been widely used to simulate shoulder activities. Few studies have reported on in-vivo shoulder biomechanics due to the complicated shoulder anatomy and limitations of quantitative measurement methods. In this study, we utilized a validated DFIS and MR imaging technique to investigate the 3D kinematics of the humeral head and the elongation patterns of the glenohumeral ligaments.

The data demonstrated that during abduction motion of the shoulder, the humeral head center translated in the anterior-posterior direction in a range of 6.0mm and in the superior-inferior direction in a range of 2.5mm. Furthermore, the humeral head was compressed towards the glenoid by an average of 1.6mm as compared to the resting position. These data may indicate that the laxity of the shoulder joint is higher in the anterior-posterior direction than the other two directions for the in-vivo shoulder positions studied, further supporting the concept that normal shoulder joint motion is not ball-in-socket kinematics as suggested by some prior studies [11,16,25-27].

Inconsistent humeral head kinematics have been reported in the literature. Bigliani et al. has reported [28] minimal translations in all three anatomic directions during abduction in the scapular plane using cadaveric specimens. Harryman et al. has reported [11] posterior translation during extension and external rotation, and anterior translation during internal rotation and cross-body movement during passive glenohumeral motion using cadaveric specimens. Wuelker et al. has reported 9.0 ± 5.2mm superior and 4.4 ± 1.3mm anterior translation during 20° to 90° of simulated elevation [27]. In general, our data showed a larger anterior-posterior motion range than those reported in the literature. These variations in kinematics may be due to differences in experimental setup as well as the simulated shoulder motions in these studies. In our study, we investigated young and healthy living shoulders, whereas the specimens used in in-vitro studies are typically from older donors. In addition, most in-vitro studies dissect the shoulder down to the capsule level and this may alter the kinematic nature of the joint.

The SGHL elongated the most at 45° and further abduction decreased its elongation and kept a similar length as that at 0° abduction. The data indicated that the SGHL might only function from 0° to the middle range of abduction of the shoulder. The MGHL length was minimum at 0° abduction and 90° abduction with maximum internal rotation. The MGHL elongation was about 64% at all other positions of the shoulder. Therefore, the MGHL seems to function over a broader range of shoulder motion than the SGHL. Similar observations have been reported by Warner et al. in a cadaveric study [7].

The AB-IGHL showed consistent elongation with abduction and with external rotation. The AB-IGHL elongated the most with external rotation. This observation supports both experimental and clinical observations that this structure is most important to restrain anterior subluxation of the humeral head during the externally rotated throwing position. The PB-IGHL showed a different elongation pattern than the AB-IGHL. The PB-IGHL demonstrated moderate elongation at 90° abduction and with external rotations, though it showed maximum elongation at 90° abduction with maximum internal rotation. There appeared to be a reciprocal function between the AB-IGHL and PB-IGHL during in-vivo shoulder motion, similar to the mechanism proposed by O’Brien et al. and Warner et al. in cadaveric studies [2,3]

The ultimate strain [8-10] of the ligaments of the shoulder are in the range of 7 to 36%. Therefore, the large in-vivo elongations of the ligaments measured in this study may indicate that the glenohumeral ligaments only function in the positions where they show maximum elongation. Effectively, the glenohumeral ligaments are lax in most of the functional range of the shoulder, as this appears to be a necessary feature for normal shoulder mechanics, so as to not over-restrain the joint. The muscular contraction, ligament wrap lengths and articular geometry of the glenohumeral joint are thought [6] to be the major stabilizing factors of the shoulder, whereas the glenohumeral joint capsule provides passive stability at the extremes of glenohumeral motion [24]. For example, the SGHL may only function below the middle range of abduction, while the MGHL may function from the middle range of abduction to 90° abduction with maximum external rotation. The AB-IGHL may function at high abduction angles with external rotation while the PB-IGHL may function at high abduction angles with internal rotation of the shoulder.

It should be restated that this study evaluated the relative elongation of the glenohumeral ligaments using 0° abduction ligament lengths as the reference. The DFIS and MR modeling technique were unable to account for any laxity in the measurement of the actual in-vivo glenohumeral ligaments lengths. Therefore, it is difficult to extract ligament strain values from these data. A future anatomic study should examine the zero loading stretched length of the ligaments so that in-vivo shoulder ligament strain can be quantified. Nonetheless, this study provided quantitative data on the range of humeral head motion and ligament elongations during functional in-vivo shoulder positions. The length patterns of the surrounding ligaments may also provide a reference point for surgical tightening after injury of the ligaments so that normal shoulder kinematics can be restored. No external loads other than the forearm weight were considered in this study. All shoulders were investigated under static arm positions. With further development of the image acquisition and data processing techniques, shoulder biomechanics under dynamic conditions should be investigated. This study also only investigated normal shoulder biomechanics without distinguishing gender and dominance differences. In order to delineate gender and dominance effects, more subjects would need to be investigated. Nevertheless, the data obtained in this study were compared to previous literature with good agreement, and indirectly validated the DFIS and MR modeling technique for glenohumeral ligament measurements.

## Conclusion

This study investigated the humeral head translations and shoulder glenohumeral ligaments elongations during in-vivo activities using a combined DFIS and MR imaging technique. The results demonstrated that the humeral head translated statistically more in the anterior-posterior than the superior-inferior direction during abduction and rotation of the shoulder, which supports the concept that glenohumeral kinematics are not ball-in-socket mechanics. The SGHL may only function at low abduction angles of the shoulder and the MGHL seems to function above 45° of abduction and over a broader range of shoulder motion than the SGHL. The AB-IGHL may be most important to restrain anterior subluxation of the humeral head during the externally rotated throwing position. The PB-IGHL showed maximum elongation at 90° abduction with maximum internal rotation. There appeared to be a reciprocal function between the AB-IGHL and PB-IGHL during in-vivo shoulder motion. In this study, we have shown that shoulder ligaments function in different ranges of shoulder positions. These data suggest that in healthy adult shoulders the ligamentous structures of the glenohumeral joint are not fully elongated in many shoulder positions, but function as restraints at the extremes of glenohumeral motion. Clinically, these results may be helpful in restoring ligament anatomy during the treatment of anterior instability of the shoulder.

## Competing interests

The author(s) declare that they have no competing interests.

## Authors’ contributions

DFM: analyzed humeral head translations and ligament elongations, drafted/revised manuscript, PJB: recruited subjects, organized MRIs, took fluoroscopic images, analyzed humeral head translations and ligament elongations, drafted manuscript, RP: analyzed humeral head translations and ligament elongations, drafted manuscript, TJG: revised manuscript and gave final approval, JPW: revised manuscript and gave final approval, GL: conceived of the study, revised manuscript and gave final approval, All authors read and approved the final manuscript.
